# From eels to the importance of cancer biobanks

**DOI:** 10.4155/fso.15.65

**Published:** 2015-11-01

**Authors:** Paul N Span

**Affiliations:** 1 Radboud University Nijmegen Medical Centre, The Netherlands

**Keywords:** Anguilla anguilla, biobanks, oncology

Paul Span obtained his PhD on steroid metabolism from the Radboud University Nijmegen in 1996. He is currently Associate Professor at the Department of Radiation Oncology. Here, the experimental and clinical research is aimed at molecular and functional imaging of tumors at the macroscopic and microscopic level in head and neck and breast cancer, and more recently colorectal, lung and prostate cancer. An important objective is the development of predictive profiles based on microenvironmental tumor characteristics to ultimately provide a mechanistic basis for the optimization of treatments that combine radiotherapy with novel biological modifiers and for development of patient selection strategies. He is Basic Affairs Officer and member of the Steering Committee of the PathoBiology Group of the European Organisation for Research and Treatment of Cancer.

## Q Can you tell us a little about your career to date & how you came into oncology?

I studied Biology and actually did my master thesis on steroid metabolism in eels. At that moment I knew I would rather continue my career investigating human subjects, and was lucky enough to find a position at the Department of Chemical Endocrinology of the Radboud University Medical Center in Nijmegen (The Netherlands). After my PhD on prostate steroid metabolism, we started exploiting a large breast tumor biobank that was available at the department, under the direction of professor Fred Sweep. This biobank contained thousands of breast cancer tissues that had remained after measuring estrogen receptor content for treatment selection for these patients. A significant proportion of patients was treated with surgery, with or without radiotherapy, but did not receive endocrine or chemotherapy. This made this cohort ideal for studies into prognostic factors, in other words, factors that might predict how the disease will progress irrespective of adjuvant treatment. Most of the prognostic and predictive factors we have since identified turned out to be hypoxia related, eventually leading to me now working at the Radiation Oncology department, which has a long-lasting expertise in hypoxia.

## Q What are you working on at the moment?

Our main interest at the moment is in how tumor cells are capable of surviving hypoxia, more specifically the unfolded protein response, and autophagy. It is striking how tumor cells develop mechanisms to counter the hostile microenvironment. The tumor cells in hypoxic regions are also very resistant to radio- and chemotherapy. If we would be able to counter the hypoxia resistance mechanisms of tumor cells, these cells would die, leaving tumor cells that are relatively easy to target [1]. We are venturing into a number of – for us – new subjects, such as intracellular signaling pathways [2] and metabolism [3]. I think it is one of the great things about research that you will always be learning new things. I am also very happy with the combination of clinical, translational and basic research that I am able to do because of the location of the laboratory within the clinical Radiation Oncology department, and because of the excellent working relationship with the radiation oncologist and head of the laboratory Dr Bussink.

## Q You are involved with the Radboud & Nijmegen Breast Cancer Biobanks – can you tell us a little about these?

As mentioned before, I started working in oncology mainly because of the availability of a large clinical breast cancer tissue biobank. This was, however, a retrospective biobank of breast cancer patients for which it was necessary to measure the estrogen receptor (which leads to some bias), and who were treated very much differently compared with current regimens. I have been working on setting up a prospective collection of biomaterial, clinical data and informed consent from all breast cancer patients at our medical center for some time, and we started doing this over a year ago. This was possible because the Radboudumc initiated the Radboud biobank, facilitating much of the infrastructure, and because a former PhD student of mine, Dr Manders, who now leads this biobank.

## Q What are you doing currently with the International Cancer Genome Consortium?

We contribute to the International Cancer Genome Consortium (ICGC) in the collection of breast cancer samples for whole genome and RNA sequencing, methylation analyses and such. This work has already been very fruitful, generating papers in high-ranking journals [4,5], with many more publications to come. It is an example of how important large, multinational, well-organized biobanks are. These biobanks will also prove to be invaluable for future research, clinically or independently validating data from other studies.

## Q Can you tell us a little about your role with the European Organisation for Research & Treatment of Cancer?

My work within the European Organisation for Research and Treatment of Cancer is performed within the PathoBiology Group (PBG), which was founded – as the Receptor and Biomarker Group – many years ago. The PBG is focused on linking translational research group with clinical groups, for identification and validation of clinical biomarkers. Most of the people within this group have known each other for many years, making it very easy to set up collaborations for joint research.

## Q What do you think are the biggest challenges for personalized medicine in oncology at the moment?

The concept of personalized medicine is an important, but considering the temporal and inter- and intra-tumor heterogeneity, difficult-to-obtain goal. Despite our recent advances in understanding the effect of genomic instability, Darwinian selection of treatment-resistant clones, the effect of the tumor microenvironment and so on, most treatment regimens are given per protocol to large numbers of cancer patients irrespective of their genotypes and phenotypes.

## Q If you could, how would you go about addressing this?

Any advance we wish to make will depend on well-organized biobanks and trials, and the public availability of all data that is subsequently obtained. Thus, I believe it is crucial that biomaterials, clinical data and informed consent are obtained of every patient, not only in trials but also in a ‘routine’ setting. Therefore I am trying to extend our biobank initiative to other nonacademic hospitals within our region. Furthermore, I applaud open access journal initiatives such as *Future Science OA*. It will be absolutely crucial that all data are available for all researchers if we are to move forward.

## Q How do you see personalized medicine in oncology changing over the next 10 years?

Only a few biomarkers for selection of cancer patients for particular treatments have so far made it to the clinic. I think we need to focus on not only identifying yet more biomarkers, but also on using these in the clinic. Furthermore, we need to stay realistic in our prediction of the future, as researchers have promised the eradication of cancer several times in the past decades. I think we will certainly have made progress in better selecting the right treatment for the right patient.

## Q Finally, if you had unlimited resources at your disposal, what one piece of research would you do, & why?

As might be expected considering what we talked about, I believe biobanking to be crucial. I would be very much in favor of a continuation and extension of the ICGC initiative, for instance, leading to a worldwide, well-defined biobank that is available for all researchers. Furthermore, although not something that I do, I believe we should make much more effort in actually using newly defined biomarkers in the clinic. Close collaborations between (eel?) biologists and clinical doctors will remain necessary.
